# Long-Term Surgical Outcomes in Double Outlet Right Ventricle Based on Detailed Anatomical Sub-Typology

**DOI:** 10.1093/ejcts/ezaf334

**Published:** 2025-10-07

**Authors:** Kunjing Pang, Keming Yang, Rong Wang, Kai Ma, Nan Xu, Jiayi Xing, Li Zhang, Tingting Zhang, Shoujun Li

**Affiliations:** Department of Echocardiography, Fuwai Hospital, National Center for Cardiovascular Disease, Chinese Academy of Medical Sciences and Peking Union Medical College, Beijing 100037, China; Department of Cardiovascular Surgery, Fuwai Hospital, National Center for Cardiovascular Disease, Chinese Academy of Medical Sciences and Peking Union Medical College, Beijing 100037, China; Department of Echocardiography, Fuwai Hospital, National Center for Cardiovascular Disease, Chinese Academy of Medical Sciences and Peking Union Medical College, Beijing 100037, China; Department of Cardiovascular Surgery, Fuwai Hospital, National Center for Cardiovascular Disease, Chinese Academy of Medical Sciences and Peking Union Medical College, Beijing 100037, China; Department of Echocardiography, Fuwai Hospital, National Center for Cardiovascular Disease, Chinese Academy of Medical Sciences and Peking Union Medical College, Beijing 100037, China; Department of Echocardiography, Fuwai Hospital, National Center for Cardiovascular Disease, Chinese Academy of Medical Sciences and Peking Union Medical College, Beijing 100037, China; Department of Echocardiography, Fuwai Hospital, National Center for Cardiovascular Disease, Chinese Academy of Medical Sciences and Peking Union Medical College, Beijing 100037, China; Department of Echocardiography, Fuwai Hospital, National Center for Cardiovascular Disease, Chinese Academy of Medical Sciences and Peking Union Medical College, Beijing 100037, China; Department of Cardiovascular Surgery, Fuwai Hospital, National Center for Cardiovascular Disease, Chinese Academy of Medical Sciences and Peking Union Medical College, Beijing 100037, China

**Keywords:** double outlet right ventricle, anatomical subclassification, surgery, prognosis

## Abstract

**Objectives:**

Long-term surgical outcomes for double outlet right ventricle (DORV) continue to pose challenges for most paediatric cardiac centres. This study aimed to investigate the mid- to long-term outcomes of a large cohort of DORV patients.

**Methods:**

DORV patients who underwent surgery guided by a refined anatomical sub-typology in our centre between August 2001 and December 2023 were retrospectively reviewed. The primary end-point was cardiac mortality and the secondary end-point was unplanned surgical reintervention.

**Results:**

A total of 1135 patients were included in the study. Among them, 824 patients (72.6%) underwent biventricular repair, 258 (22.7%) received single-ventricle procedures, and 53 (4.7%) underwent palliative surgeries. The median age at surgery was 25.0 months [IQR: 9.0-72.0]. In-hospital deaths occurred in 14 cases (1.2%). Over a mean follow-up period of 8.9 years, the estimated overall survival rates at 5, 10, and 15 years were 96.3% [95% CI, 95.1-97.5], 93.0% [95% CI, 91.6-95.2], and 92.7% [95% CI, 87.7-96.9], respectively. Second end-point-free survival rates at the same time points were 95.2% [95% CI, 94.8-95.7], 89.5% [95% CI, 88.7-90.3], and 82.1% [95% CI, 80.7-83.5], respectively. Transposition of the great arteries-type (TGA-type) anatomy and palliative procedures were independently associated with increased mortality.

**Conclusions:**

Optimal outcomes in DORV can be achieved through precise anatomical evaluation and tailored surgery. TGA-type anatomy and palliative procedure were key risk predictors.

## Graphical Abstract

**Figure:**
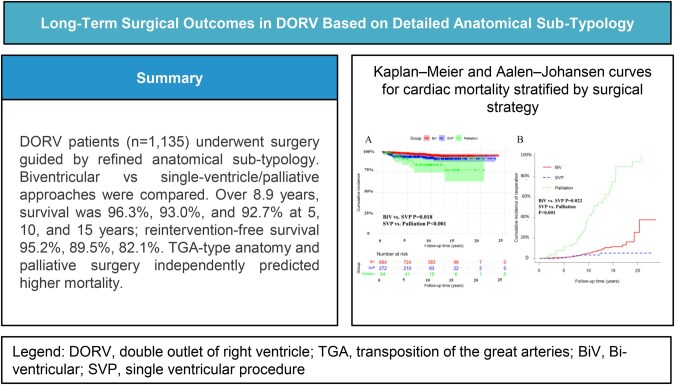

## INTRODUCTION

Double outlet right ventricle (DORV) is a complex heart defect characterized by both the pulmonary artery and aorta originating predominantly or entirely from the right ventricle.^1–4^ This condition encompasses a wide range of heart structures, showcasing diverse morphological characteristics, connections, and relationships across different cardiac segments and their junctions. Consequently, its clinical presentation and surgical interventions required vary significantly.^3^ Systematic classification remains crucial for the effective diagnosis and surgical treatment of DORV patients. The most recent classification of DORV was introduced in 2000 by the American Society of Thoracic Surgeons (STS) and the European Association for Cardio-Thoracic Surgery (EACTS) as part of the Congenital Heart Disease Nomenclature & Database Project.^1^ However, accurately delineating the anatomical features and determining the optimal surgical strategy—particularly for transposition of the great arteries (TGA)-type and remote-type DORV—remains a significant challenge at our centre. To address this, our team refined the classification of TGA-type and remote-type DORV by introducing additional subtypes within each category. Furthermore, we developed tailored surgical approaches for each subtype, enabling more precise diagnoses and optimized treatment strategies for all DORV patients.

Advances in diagnostics and surgery have significantly reduced early and in-hospital mortality in DORV patients. However, mid-to-long-term outcomes remain less favourable, with survival beyond 5 years below 90% and reoperation-free rates even lower.^5–7^ Complex anatomical variations—such as diverse subtypes and challenging intracardiac baffle pathways—continue to pose significant difficulties in the surgical management of DORV. In this study, we retrospectively demonstrated favourable surgical outcomes in a large cohort of DORV patients at our centre according to a detailed subclassification, and analysed the key contributing factors.

## METHODS

### Patient population

This single-centre retrospective study included all consecutive DORV patients who underwent surgery at Fuwai Hospital between August 2001 and December 2023. Medical records were reviewed for demographic data, diagnosis, and surgical details. Patients were eligible if they had a DORV diagnosis and received surgical treatment at our centre, including those who had initial procedures elsewhere but underwent Biventricular (BiV) repair, 1.5-ventricle repair, Fontan, or reintervention at our hospital. Exclusion criteria include (1) no surgical treatment despite imaging-confirmed DORV (*n* = 183); (2) lost to follow-up (*n* = 85); and (3) DORV with intact ventricular septum (*n* = 55).^8^ The study was approved by the Fuwai Hospital Ethics Committee, with informed consent waived due to its retrospective nature.

### Diagnosis and detailed anatomical classification of DORV

**Figure 1.**
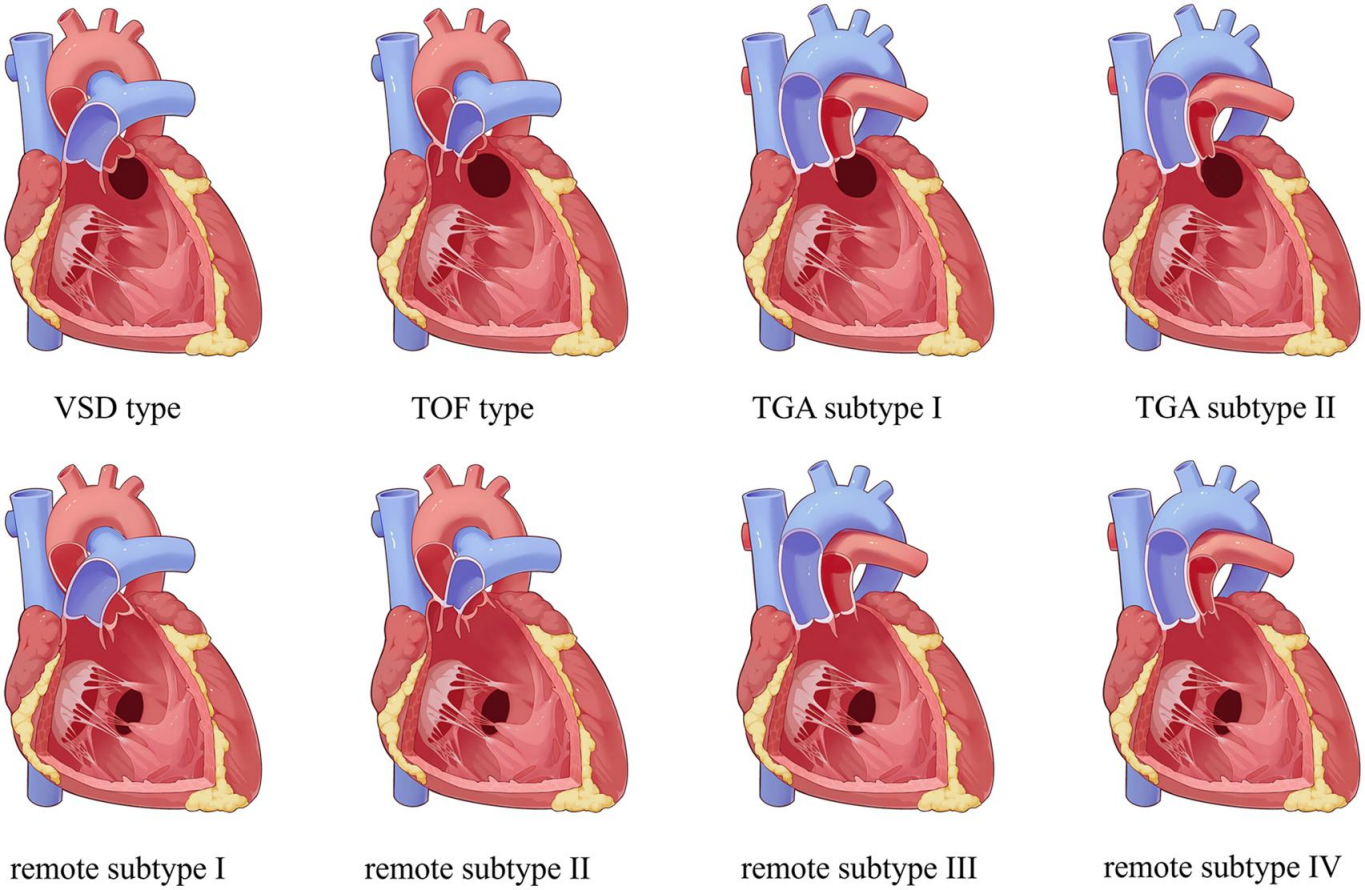
Illustration of the Refined Anatomical Subclassification. VSD, ventricular septal defect; TGA, transposition of the great arteries

The diagnosis of DORV is initially proposed through echocardiography, with multimodal imaging used to assist in diagnosis as needed, including cardiac computed tomography, angiography, or 3D type according to STS-EACTS classification.^1^ For the TGA-type and remote-type categories, we introduced additional subtypes within each category (**Table 1**, **Figure 1**). The TGA-type was subdivided into 2 subtypes (subtypes I and II) based on the presence or absence of pulmonary outflow tract obstruction (POTO). The remote-type was further divided into 4 subtypes (subtypes I-IV) according to the relationship of the great arteries and the presence or absence of POTO.^9^ Representative echocardiographic images of the all the anatomical subtypes and their corresponding postoperative appearances were shown in **Figure S2** and **Video S1**. The refined subclassification was applied retrospectively to cases treated before May 2007 and prospectively to cases treated from that time onward.

**Table 1.** Detailed Anatomical Subtypes and Corresponding Surgical Options for DORV

STS-EACTS	Detailed anatomical subtypes				Surgical options	
Anatomical type	Anatomical subtype	Relationship of great arteries	VSD location	POTO	Operational type	Procedure
VSD type		Normal	Committed	Absent	VSD type	IVR-AO
TOF type		Normal	Committed	Present	TOF type	IVR-AO+RVOTR
TGA type	TGA subtype I	TGA	Committed	Absent	TGA type I	ASO+IVR-AO
	TGA subtype II	TGA	Committed	Present	TGA type II	Rastelli/REV/DRT
Remote type	Remote subtype I	Normal	Noncommitted	Absent	Remote type I	LIVR-AO
	Remote subtype II	Normal	Noncommitted	Present	Remote type II	LIVR-AO+RVOTR
	Remote subtype III	TGA	Noncommitted	Absent	Remote type III	ASO+LIVR-AO
	Remote subtype IV	TGA	Noncommitted	Present	Remote type IV	Rastelli/REV/DRT with LIVR-AO
					SVP	Fontan
					Palliation	BT or PAB

Abbreviations: STS, the Society of Thoracic Surgeons; EACTS, the European Association for Bacardi-Thoracic Surgery; VSD, ventricular septal defect; POTO, Pulmonary Outflow Tract Obstruction; OP, operational; IVR-AO, intraventricular tunnel repair to aorta; RVOTR, right ventricular outflow tract reconstruction; LIVR-AO, Long intraventricular tunnel repair to aorta; REV, Réparation à l‘Étage Ventriculaire; ASO, Arterial switch operation; DRT, Double root translocation; SVP, single ventricular procedure; BT, Blalock-Taussig shunt; PAB, pulmonary artery banding; TOF, Tetralogy of Fallot.

### Surgical strategies

Surgical strategies were planned by a multidisciplinary team, and all operations were performed by 4 to 5 experienced congenital cardiac surgeons. BiV repairs were adapted to anatomical subtypes and categorized into 8 operative types (OP types), as previously described (**Table 1**). OP type IV includes 3 subtypes^10–15^: Rastelli, Réparation à l‘Étage Ventriculaire (REV), and double root translocation (DRT). OP type VIII also includes 3 subtypes^10–15^: Rastelli with a long intraventricular tunnel to the aorta (LIVR-AO; **Figure S2**, **Video S1**), REV with LIVR-AO (**Figure S3**, **Video S2**), and DRT with LIVR-AO (**Figure S4**, **Video S3**). The Fontan procedure was classified as single-ventricle palliation (SVP). Palliative procedures also included the Blalock-Taussig (BT) shunt and pulmonary artery banding (PAB).

### Clinical follow-up

Postoperative outcomes included mortality, heart transplantation, surgical unplanned reintervention, and clinical status. The primary end-point was cardiac death or heart transplantation. The secondary end-point was unplanned surgical reintervention. Events were confirmed through medical records, clinic visits, or telephone follow-up. Overall survival was defined from the date of surgery at our centre to the occurrence of an end-point event or last follow-up.

### Statistical analysis

Categorical variables were summarized as frequencies and percentages, while continuous variables were expressed as mean (standard deviation, SD) or median with interquartile range (IQR), as appropriate. We carefully examined the extent and pattern of missing data for baseline covariates and outcomes. The proportion of missing data was low (<5%) for all key variables; thus, complete case analysis was employed, which is considered appropriate when the missing data are minimal and assumed to be missing at random. Group comparisons were conducted using the chi-squared test for categorical variables. Receiver operating characteristic (ROC) curve analysis was performed to evaluate the predictive value of anatomical subtypes and operative types for cardiac death and secondary end-points. Kaplan-Meier survival analysis, along with the log-rank test, was used to compare survival outcomes between groups. The cumulative incidence of secondary end-points was estimated using the Aalen-Johansen method, and Gray’s test was used to compare differences between groups. Patients lost to follow-up (*n* = 85) were included in the survival analysis, with their outcomes treated as censored observations. Multivariable Cox proportional hazards models were used to identify independent risk factors for cardiac death or surgical reintervention, with results reported as hazard ratios (HRs) or subdistribution hazard ratios (subHRs), along with 95% confidence intervals (CIs). All statistical analyses were performed using SPSS version 26.0 (IBM Corp.) and R version 4.3.2 (R Foundation for Statistical Computing). A 2-sided *P*-value < 0.05 was considered statistically significant.

## RESULTS

### Patient population

A total of 1135 DORV patients were included in the study. Baseline characteristics, stratified by surgical strategy, are summarized in **Table 2**. The median age at initial surgery was 25.0 months [IQR: 9.0-72.0], and 715 patients (62.6%) were male. Prior to referral, 264 patients had undergone 362 palliative procedures at other institutions, including 151 BT shunts, 132 Glenn procedures, and 79 PAB surgeries. Preoperative diagnosis primarily relied on echocardiography. cardiac computed tomography was performed in 456 patients, cardiac catheterization in 218 patients, and 3D printing was applied in 38 patients. Ultimately, 824 patients (72.6%) received BiV repair, including 21 who underwent 1.5-ventricle repair due to atrioventricular (AV) discordance and isolated dextrocardia. A total of 258 patients (22.7%) underwent SVP, including 26 scheduled for staged Fontan completion following Glenn. The remaining 53 patients (4.6%) received only palliative procedures—25 BT shunts for hypoplastic pulmonary arteries and 28 PABs due to complex anatomy precluding BiV repair. The characteristics of remote-type DORV patients were analysed absolutely and presented in **Table S1**.

**Table 2.** Patient Characteristics

	General （n = 1135）	BiV （n = 824）	SVP （n = 258）	Palliation （n = 53）	*P*
**Clinical characteristic**					
Age (m)	25.0 [9.0,72.0]	13.0 [7.0,36.0]	64.5 [33.0,116.3]	12 [5,34]	.000
Male, n (%)	715 (62.6%)	527 (63.6)	155 (59.1)	33 (62.2)	.412
NYHA≥III	44 (3.9)	30 (3.7)	12 (4.7)	2 (3.8)	.000
**Anatomical type, n(%)**					.000
VSD type	172 (15.1)	171 (20.7)	0	1 (1.9)	
TOF type	246 (21.6)	229 (27.8)	10 (3.9)	7 (13.2)	
TGA type	215 (18.9)	163 (19.7)	42 (16.3)	8 (15.1)	
Remote type	502 (44.0)	262 (31.8)	209 (80.1))	31 (58.5)	
**Complex concomitant malformations**					
CAVC	127 (11.1)	32 (3.8)	87 (33.7)	8 (15)	.000
PAA	16 (1.4)	8 (0.9)	6 (2.2)	2 (3.7)	.038
Heterotaxy	148 (13.0)	50 (6)	87 (33.2)	11 (20.7)	.000
Isomerism	41 (3.6)	8 (0.9)	30 (11.4)	3 (5.6)	.000
Straddling AV valve	21 (1.8)	9 (1.0)	9 (3.4)	3 (5.6)	.010
TAPVC	36 (3.1)	16 (1.9)	19 (7.2)	1 (1.8)	.007
AV discordance	75 (6.5)	22 (2.6)	47 (17.9)	6 (11.3)	.000
FSV	51 (4.4)	0.0 (0.0)	41 (15.9)	10 (18.8)	.000
Crisscross	20 (1.7)	4 (0.4)	15 (5.7)	1 (1.8)	.000
**History of procedure**	264 (23.2)	134 (16.2)	113 (43.7)	17 (32.0)	.000
**Follow-up**					
Number of procedures	1.3 ± 0.5	1.2 ± 0.5	1.5 ± 0.5	1.4 ± 0.6	.000
FU(y)	8.9 ± 4.6	9.2 ± 4.6	8.4 ± 4.5	7.8 [2.4,11.6]	.171
Primary end-points	62 (5.4)	33 (4.0)	19 (7.3)	9 (16.9)	.000
Second end-points	71 (6.2)	43 (5.2)	5 (1.9）	23 (43.3)	.000
In-hospital death	14 (1.2)	8 (0.9)	4 (1.5)	2 (3.7)	.175

The anatomical type is according to STS/EACTS classification in 2000; The BiV group included 15 cases of 1.5 ventricular repair.

Abbreviations: BiV, biventricle; SVP, single ventricle palliation; NYHA, New York Heart Association; AV, atrioventricular; CAVC, complete atrioventricular canal; PAA, pulmonary valve atresia; COA, coarctation of aorta; TAPVC, Total Anomalous Pulmonary Venous Connection; FSV, functional single ventricle; FU, follow-up; TOF, Tetralogy of Fallot.

### Distribution of anatomical subtypes and operational types

**Figure 2.**
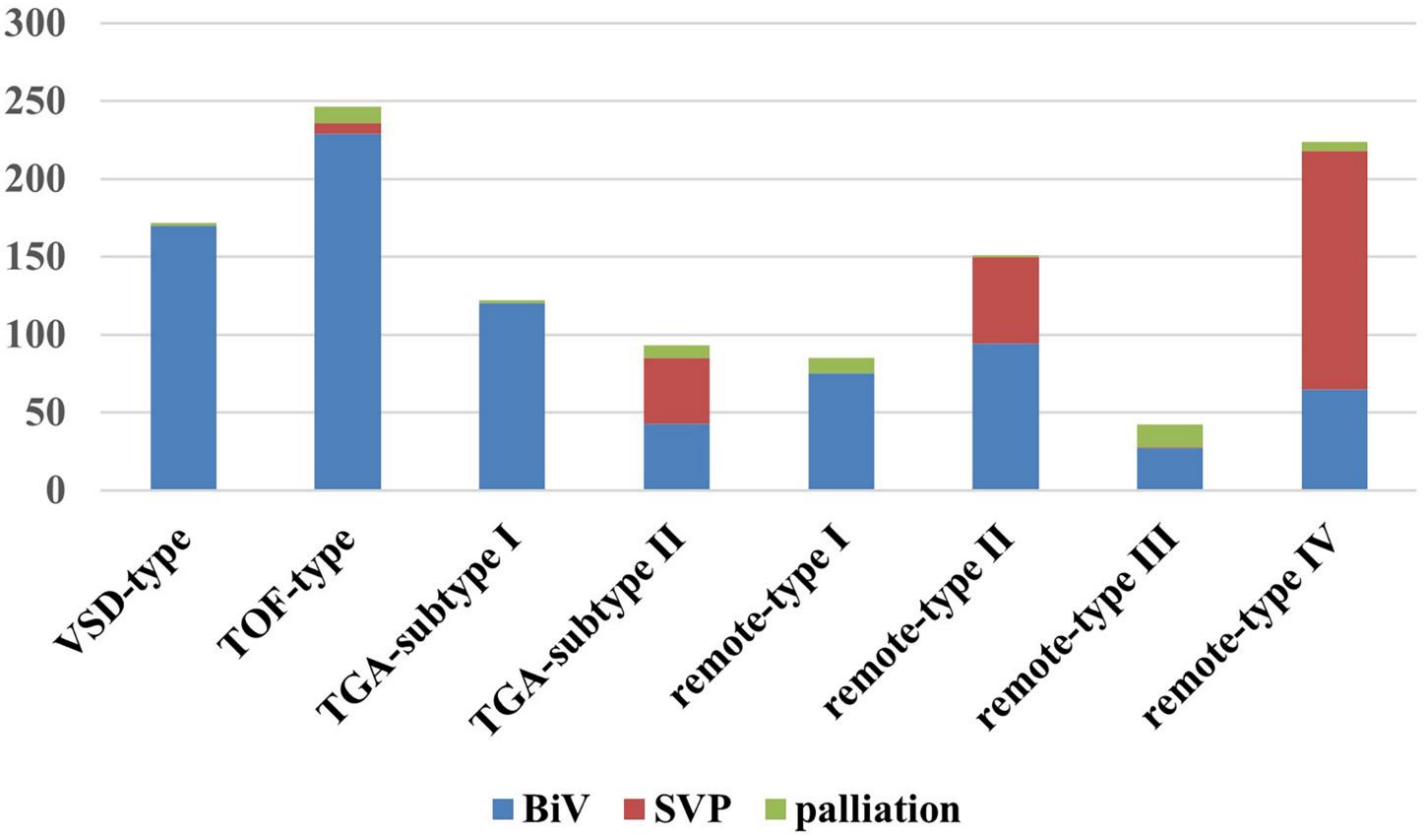
Distribution of Surgical Types Across All the Anatomical Subtypes of the Total Cohort

The distribution of anatomical subtypes and OP types are depicted in **Figure S5**. The corresponding procedures and results for the entire cohort, stratified according to the 4 anatomical types (including 4 subtypes), are illustrated in **Figure 2** and **Table S2**. Apart from cases that opted for SVP or Palliative procedures, all patients were assigned the BiV OP types corresponding to their anatomical subtypes (**Table S2**). The reasons for choosing SVP procedures for 258 patients are as follows: (1) complex concomitant malformations, including 41 cases of functional single ventricle (FSV), 15 cases of crisscross heart, 21 cases of straddling AV valve, 47 cases of AV discordance combined with heterotaxy or isomerism, and 53 cases of CAVC combined with heterotaxy, isomerism, or AV discordance, 3 cases anatomical subtype II patients had previously undergone Glenn procedures due to associated AVSD, and Fontan surgery was ultimately chosen as the definitive strategy after referral to our centre. (2) Additionally, 73 patients had remoteVSD which were evaluated as being difficult to repair through LIVR-AO procedures (**Figure S7**).

### Patient clinical outcomes

Over a mean follow-up of 8.9 years (SD 4.6), 62 patients (5.4%) reached the primary end-point, including 61 cardiac deaths and 1 heart transplant. A total of 136 patients (11.9%) met the secondary end-point, consisting of 65 all-cause deaths and 71 unplanned surgical reinterventions. In-hospital mortality occurred in 14 patients (1.2%), mainly due to refractory postoperative left heart failure or pulmonary hypertension crisis. Surgical reinterventions were most commonly performed for right ventricular outflow tract obstruction (RVOTO; 51 cases, 71.8%), followed by left ventricular outflow tract obstruction (LVOTO; 11 cases, 15.5%) and atrioventricular valve repair or replacement (9 cases, 12.7%).

For the entire cohort, estimated transplant-free survival rates at 5, 10, and 15 years were 96.3% [95% CI, 95.1-97.5], 93.0% [95% CI, 91.6-95.2], and 92.7% [95% CI, 87.7-96.9], respectively. In the overall cohort, the freedom from reoperation was 95.2% [95% CI, 94.8-95.7] at 5 years, 89.5% [95% CI, 88.7-90.3] at 10 years, and 82.1% [95% CI< 80.7-83.5] at 15 years, based on the cumulative incidence function accounting for competing risk from mortality. Survival free from end-points in Each Anatomical Subgroup Defined by the STS/EACTS Classification were illustrated in **Table S3**. Detailed survival outcomes by surgical strategy were presented in **Table S4**.

### Risk factors

**Figure 3.**
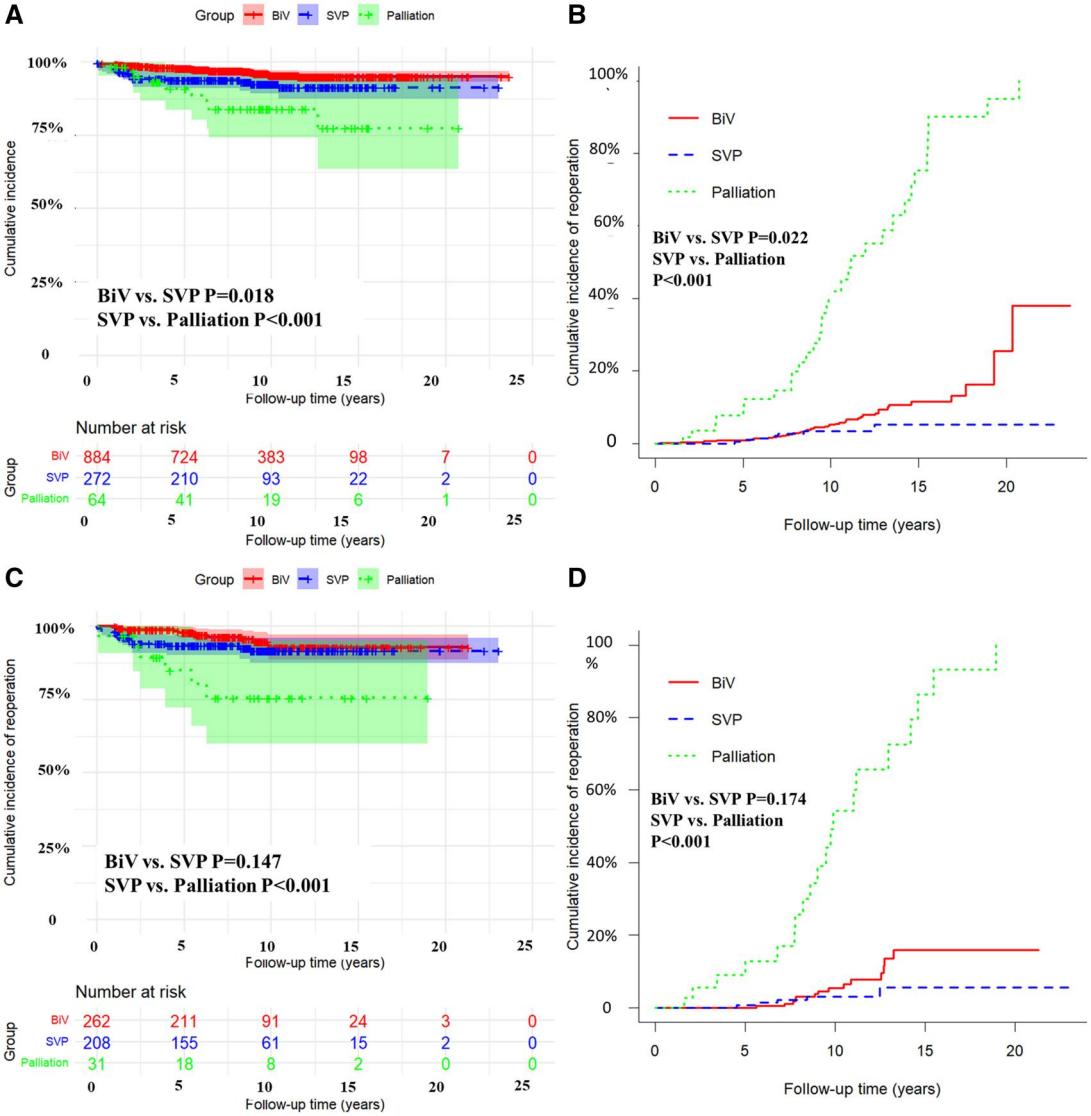
Kaplan-Meier and Aalen-Johansen Curves for Primary and Secondary End-Points. (A) Kaplan-Meier curve depicting freedom from primary end-points in the overall cohort, stratified by surgical strategy: Biventricular repair (BiV), Single Ventricle Palliation (SVP), and initial palliation. (B) Aalen-Johansen estimator for cumulative incidence of surgical reinterventions in the overall cohort. (C) Kaplan-Meier curve for freedom from primary end-points among remote-type DORV patients, stratified by surgical strategy. (D) Aalen-Johansen estimator for surgical reinterventions in remote-type DORV patients

Kaplan-Meier analysis showed that surgical strategy significantly affected long-term outcomes. The BiV group had the highest freedom from cardiac death, while the palliation group had the lowest (*P* < 0.001; **Figure 3A**). Mortality did not differ significantly before vs after 2014 (*P* = 0.600). The cumulative incidence of unplanned reoperations varied by group. Gray’s test showed significant differences between BiV and SVP (*P* = 0.022) and between SVP and palliation (*P* < 0.001; **Figure 3B**). Among patients with remote type DORV, no significant difference in cardiac outcomes was found between BiV and SVP (*P* = 0.147; **Figure 3C**), but SVP still showed better outcomes than palliation (*P* < 0.001). Similarly, reoperation rates were not significantly different between BiV and SVP (*P* = 0.174), but SVP had better outcomes than palliation (*P* < 0.001; **Figure 3D**).

Cox regression analysis identified anatomical TGA subtypes I and II, as well as palliative procedures, as independent risk factors for cardiac mortality (HR = 4.1, 95% CI: 1.1-15.4, *P *= 0.039; HR = 5.0, 95% CI: 1.3-19.8, *P *= 0.022; and HR = 3.1, 95% CI: 1.4-7.3, *P *= 0.008, respectively; **see Table S5**). Fine-Gray competing risk regression revealed that heterotaxy syndrome and palliative procedures were independently associated with a higher risk of unplanned surgical reinterventions (subHR = 1.8, 95% CI: 1.0-3.1, *P *= 0.016; and subHR = 6.7, 95% CI: 4.1-10.9, *P *< 0.001, respectively). In contrast, SVP was independently associated with a significantly lower reoperation risk (subHR = 0.2, 95% CI: 0.1-0.6, *P *= 0.002; **Table S6**).

## DISCUSSION

This study reports the mid- to long-term outcomes of a large DORV cohort treated surgically at the National Center for Cardiovascular Diseases in China over a 22-year period. The majority of patients received detailed anatomical evaluations, and surgical strategies were tailored accordingly. Favourable outcomes were achieved, leading to the following key findings: (1) Overall surgical outcomes for DORV were excellent, exceeding those previously reported; (2) Detailed anatomical subclassification was associated with appropriate selection of BiV candidates; and (3) TGA-type, as well as palliative surgery, was independently associated with increased mortality risk.

Persistent confusion in the classification and surgical management of DORV stems from its highly heterogeneous anatomy and complex associated malformations.^16–18^ While the STS and EACTS classification offers sufficient clarity for VSD-type and Tetralogy of Fallot (TOF)-type DORV, surgical planning for TGA-type and remote-type cases remains challenging.^18^ To address this, our centre developed a refined subclassification for these 2 categories and established a “one-to-one” anatomical diagnostic-surgical framework, which has significantly improved the surgical decision-making process. To our knowledge, this study presents the most favourable surgical outcomes reported to date in the largest DORV cohort.^5–7^

Despite the majority of patients undergoing the intended BiV repair, surgical decision-making remained particularly challenging in remote-type DORV cases.^19^^,^^20^ In addition to absolute contraindications—such as FSV, straddling AV valves—several anatomical complexities influenced the choice between BiV and SVP strategies. SVP was generally favoured under the following conditions: (1) Isolated levocardia or dextrocardia with AV discordance, which poses technical challenges for constructing a complete atrial baffle, as required in the standard Mustard procedure; in such cases, the 1.5 V repair may be considered, but if CAVC is present, SVP is preferred. (2) Isomerism, often accompanied by abnormal systemic/pulmonary venous return and heterotaxy, typically necessitates SVP. (3) In remote-type cases where the planned long intraventricular tunnel traverses the tricuspid inflow tract, postoperative inflow stenosis may render BiV repair unfeasible (**Figure S7 and Video S4**).

Among the entire cohort, 680 patients (82.5%) underwent primary BiV repair, while 144 (17.5%) received staged BiV repair following an initial palliative procedure. The rationale for staged repair falls into 3 categories: (1) Some patients initially considered for SVP were later referred to our centre for BiV conversion. (2) In infants under 6 months with complex anatomy—such as challenging AV valve morphology or baffle pathways—an initial palliative procedure (eg, BT shunt or PA banding) was preferred to mitigate surgical risk, with definitive BiV repair planned at 1-2 years of age. (3) A subset of patients had underdeveloped pulmonary arteries requiring initial Sano or BT shunts before BiV repair. This staged approach, along with 23.2% of patients having prior palliative procedures at other institutions, contributed to a relatively older age at surgery in our cohort, particularly among SVP patients, compared to previously reported series.^5–7^

The general cohort demonstrated that BiV repair results in higher survival rates compared to SVP and palliative procedures. However, there is no significant difference in incidence of reinterventions between the BiV repair and SVP groups. The main reason is that the BiV repair group need more unplanned reinterventions than SVP group (5.2% vs 1.9%). Among them, reinterventions for RVOTO constituted the majority (71.8%), consistent with the findings reported by Oladunjoye et al.^7^ Among patients requiring right ventricular outflow tract (RVOT) reconstruction, regardless of the material used, some patients experienced early pulmonary valve restenosis or severe regurgitation. The number of unplanned reinterventions for LVOTO remains relatively low (15.5%), primarily due to the selection of patients under 6 months of age with complex anatomical subtypes for a staged surgical approach. Generally speaking, BiV repair is undoubtedly the more optimal option for DORV patients. However, SVP procedure may sometimes be a rational choice for those with excessively complicated anatomy.

This study also explored risk factors influencing surgical outcomes in patients with DORV. Palliative surgery was strongly associated with poor outcomes, highlighting the importance of early intervention to facilitate the possibility of BiV repair. TGA-type was identified as independent predictors of cardiac mortality. Both early and late deaths were often linked to preoperative coronary anomalies which posed significant challenges for coronary implantation and led to postoperative coronary artery stenosis as a major cause of mortality. TGA-type subtypes I and II demonstrated the highest incidence of such anomalies.

Severe pulmonary hypertension was another key contributor to postoperative cardiac death, especially in patients with TGA subtype I who underwent surgery after 6 months of age. Notably, remote subtypes—despite requiring technically demanding intracardiac conduit procedures—were not associated with increased mortality. This may be attributed to comprehensive preoperative echocardiographic evaluations, which allowed for accurate assessment of anatomical feasibility and careful selection between BiV repair and SVP. Additional factors likely contributing to favourable outcomes include the consistency and expertise of our dedicated surgical team, as well as the use of advanced perioperative support such as extracorporeal membrane oxygenation (ECMO) for critically ill patients.

### Limitations

This was a retrospective observational study conducted at a national cardiovascular centre in China, and certain limitations should be noted. First, potential selection bias exists, and the patient population may not fully represent the broader DORV population. Some patients had undergone prior procedures and were referred to our centre for BiV repair or reintervention, which may have influenced the outcomes. Additionally, the cohort presented at an older age compared to those in previous studies, which could suggest a generally healthier population, as more severe cases may have died before referral. Lastly, the study only included patients who underwent surgical treatment; those who did not receive surgery—often representing more severe or inoperable cases—were excluded, potentially limiting the generalizability of the findings.

## CONCLUSIONS

Overall surgical outcomes for DORV were optimal. A detailed anatomical subclassification system may facilitate individualized surgical decision-making in DORV.

## Supplementary Material

ezaf334_Supplementary_Data

## Data Availability

Data supporting the conclusions of this study are either contained within the paper or are available from the authors on reasonable request.
