# Supplementary material for: Long-Term Surgical Outcomes in Double Outlet Right Ventricle Based on Detailed Anatomical Sub-Typology
Source: Eur J Cardiothorac Surg. 2025 Oct 7;67(10):ezaf334. doi: 10.1093/ejcts/ezaf334 (PMC12553358; doi:10.1093/ejcts/ezaf334)
Supplement: ezaf334_Supplementary_Data [file ezaf334_supplementary_data.zip › Suplementary material.docx]

Two key anatomical criteria essential for the diagnosis of DORV

1) For the diagnosis of DORV, the extent of arterial override or predominant RV origin of the arteries is crucial. When the arterial relationship is normal (Nor-A) and continuity between the aorta and the mitral valve is present, an override of the aorta by ≥75% is required for a DORV diagnosis. However, if there is a discontinuity between the aorta and the mitral valve, an aortic override of ≥50% suffices for diagnosis (Supplemental Figure 1). Conversely, in cases where the arterial relationship is TGA-type, a pulmonary artery origin from the RV of ≥50% is adequate for a DORV diagnosis (Supplemental Figure 2), regardless of whether pulmonary-to-mitral valve continuity exists. 2) An non-committed ventricular septal defect is defined when the straight-line distance from the center of the VSD to the center of the semilunar valve annulus measured by echocardiography exceeds the matched diameter of the aortic valve annulus^5^. If this condition is not met, the defect is classified as a committed VSD (VSD-C).

**Supplemental Table 1** **Characteristics of remote-type DORV patients**

|  | **general （n=447）** | **BiV （n=255）** | **SVP （n=167）** | **palliation （n=25）** | **p** |
| --- | --- | --- | --- | --- | --- |
| **Clinical characteristic** |  |  |  |  |  |
| age(m) | 70.0±81.4 | 63.9±82.6 | 84.4±81.3 | 36.5±45.1 | 0.004 |
| male n(%) | 282（63.1%） | 163（63.9%） | 99（59.3%） | 20（80%） | 0.123 |
| height | 97.0（41.0） | 97.0±30.5 | 110.4±30.1 | 83.5±27.6 | 0.000 |
| weight | 18.6±14.9 | 17.2±14.2 | 21.8±15.9 | 12.0±10.2 | 0.000 |
| SBP(mmHg) | 100.0±13.3 | 98.4±12.5 | 104.0±14.2 | 93.5±9.8 | 0.002 |
| DBP(mmHg) | 60.5±11.8 | 58.9±11.5 | 63.7±12.3 | 58.1±9.9 | 0.016 |
| C/T | 0.56（0.12） | 0.58±0.08 | 0.52±0.09 | 0.59±0.08 | 0.000 |
| QRS(ms) | 91.4±24.2 | 94.7±26.4 | 86.3±20.5 | 90.3±15.1 | 0.012 |
| LVEF(%) | 65.4±7.5 | 66.0±6.9 | 64.6±7.3 | 64.4±12.8 | 0.152 |
| SO2(%) | 80.4±11.3 | 82.4±13.1 | 77.1±7.1 | 81.5±10.8 | 0.000 |
| Hb(g/dl) | 156.1±58.9 | 147.3±69.8 | 171.0（50.0） | 137.0±22.0 | 0.000 |
| **sub-type n(%)** |  |  |  |  |  |
| I | 82（18.3%） | 74（29.0%） | 0 | 8（32.0%) | 0.000 |
| II | 147（32.9%） | 94（36.9%） | 52（31.1%） | 1（4.0%） | 0.003 |
| III | 34（7.6%） | 24（9.4%） | 0 | 10（40.0%） | 0.000 |
| IV | 184（41.2%） | 63（24.7%） | 115（68.9%） | 6（24.0%） | 0.000 |
| **complex concomitant** |  |  |  |  |  |
| CAVC | 86（19.2%） | 16（6.3%） | 66（39.5%） | 4（16.0%） | 0.000 |
| PAA | 7（1.6%） | 4（1.6%） | 3（1.8%） | 0 | 1.000 |
| Heterotaxy | 88（19.7%） | 23（9.0%） | 59（35.3%） | 6(24.0) | 0.000 |
| Isomerism | 26（5.8%） | 5（2.0%） | 19（11.4%） | 2（8.0%） | 0.000 |
| staddling AV valve | 0 | 0 | 0 | 0 |  |
| COA | 4（0.9%） | 4（1.6%） | 0 | 0 | 0.330 |
| TAPVC | 19（4.3%） | 6（2.4%） | 13（7.8%） | 0 | 0.014 |
| AV discordance | 39（8.7%） | 12（4.7%） | 24（14.4%） | 3（12.0%） | 0.002 |
| FSV | 0 | 0 | 0 | 0 |  |
| Crisscross | 0 | 0 | 0 | 0 |  |
| **procesure** |  |  |  |  |  |
| CPB(min) | 147.6±79.2 | 173.3±73.5 | 130.4±63.2 | 0 | 0.000 |
| ACC(min) | 95.3±54.8 | 116.3±47.4 | 69.9±41.0 | 0 | 0.000 |
| VT(h) | 80.0±210.8 | 93.0±214.2 | 50.5±18.17 | 148.6±323.7 | 0.067 |
| ICU(h) | 169.6±334.1 | 194.8±374.2 | 124.0±240.3 | 219.1±414.1 | 0.078 |
| **follow up** |  |  |  |  |  |
| LVEF1 | 62.9±6.9 | 63.3±6.3 | 62.0±7.7 | 65.6±7.5 | 0.046 |
| NYHA1 |  |  |  |  |  |
| I | 336（75.2%） | 244（95.7%） | 89（53.3%） | 3（12.0%） | 0.000 |
| II | 87（19.5%） | 7（2.7%） | 71（42.5%） | 9（36.0%） | 0.000 |
| III | 22（4.9%） | 3（1.2%） | 6（3.6%） | 13（52.0%） | 0.000 |
| IV | 1（0.2%） | 0 | 1（0.6%） | 0 | 0.430 |
| number of operations |  |  |  |  |  |
| 1 | 300(67.1%) | 191(74.9%) | 92（55.1%） | 17(68.0%) | 0.000 |
| 2 | 132（29.5%） | 53（20.8%） | 72（43.1%） | 7（28.0%） | 0.000 |
| ≧3 | 15（3.4%） | 11（4.3%） | 3(1.8%) | 1（4.0%） | 0.367 |
| FU(y) | 8.3±4.2 | 8.5±4.2 | 8.2±4.6 | 7.5±5.1 | 0.479 |
| primary endpoints | 31(6.9%) | 12(4.7%) | 15(9.0%) | 4(16.0%) | 0.044 |
| Unplanned reintervention | 63(14.1%) | 25(9.8%) | 22(13.2%) | 9(36.0%) | 0.000 |

Abbreviations as in Table 2.

**Supplemental Table 2 Corresponding procedures and results for the entire cohort, stratified according to the eight anatomical subtypes**

|  | VSD type (n=172) | TOF type(n=246) | TGA subtype I (n=122) | TGA subtype II (n=93) | remoted subtype I (n=85) | remoted subtype II (n=151) | remoted subtype III (n=42) | remoted subtype IV VIII (n=224) | p |
| --- | --- | --- | --- | --- | --- | --- | --- | --- | --- |
| **n (%)** | 172(15.1) | 246(21.6) | 122(10.7) | 93(8.1) | 85(7.4) | 151(13.3) | 42(3.7) | 224(19.6) | 0.000 |
| **complex concomitant malformations** |  |  |  |  |  |  |  |  |  |
| CAVC n (%) | 5(2.9) | 10(4.0) | 1(0.8) | 7(7.5) | 5(5.8) | 40(26.4) | 2(4.7) | 57(25.4) | 0.000 |
| PAA | 0 | 5(2.0) | 0 | 4(4.3) | 0 | 1(0.6) | 0 | 6(2.6) | 0.030 |
| Heterotaxy | 7(4.0) | 12(4.8) | 4(3.2) | 24(25.8) | 7(8.2) | 27(17.8) | 4(9.5) | 63(28.1) | 0.000 |
| Isomerism | 1(0.5) | 1(0.4) | 0 | 7(7.5) | 1(1.1) | 5(3.3) | 0 | 26(11.6) | 0.000 |
| straddling AV valve | 4(2.3) | 3(1.2) | 0 | 2(2.1) | 1(1.1) | 1(0.6) | 4(9.5) | 6(2.6) | 0.008 |
| TAPVC | 6(3.4) | 2(0.8) | 1(0.8) | 7(7.5) | 1(1.1) | 5(3.3) | 1(2.3) | 13(5.8) | 0.009 |
| AV discordance | 2(1.1) | 1(0.4) | 4(3.2) | 21(22.5) | 1(1.1) | 7(4.6) | 5(11.9) | 34(15.1) | 0.000 |
| FSV | 2(1.1) | 7(2.8) | 1(0.8) | 9(9.6) | 1(1.1) | 3(1.9) | 4(9.5) | 24(10.7) | 0.000 |
| Crisscross | 1(0.5) | 0 | 1(0.8) | 3(3.2) | 1(1.1) | 0 | 1(2.3) | 13(5.8) | 0.000 |
| **OP type n(%)** |  |  |  |  |  |  |  |  |  |
| OP typeI | 170(98.8) |  |  |  |  |  |  |  |  |
| OP typeII |  | 229(93.0) |  |  |  |  |  |  |  |
| OP typeIII |  |  |  |  | 75(88.2) |  |  |  |  |
| OP typeIV |  |  |  |  |  | 94(62.3) |  |  |  |
| OP typeV |  |  | 120(98.3) |  |  |  |  |  |  |
| OP typeVI |  |  |  | 43(46.2) |  |  |  |  |  |
| OP typeVII |  |  |  |  |  |  | 27(64.2) |  |  |
| OP typeVIII |  |  |  |  |  |  |  | 65(29.2) |  |
| SVP |  | 7(2.8) |  | 42(45.2) |  | 56(37.1) | 1(2.3) | 153(68.2) |  |
| Palliation | 2(1.2) | 10(4.2) | 2(1.7) | 8(8.6) | 10(11.8) | 1(0.6) | 14(33.3) | 6(2.6) |  |
| Number of surgeries | 1.4±0.5 | 1.4±0.5 | 1.1±0.3 | 1.4±0.5 | 1.3±0.6 | 1.4±0.5 | 1.2±0.5 | 1.5±0.6 | 0.000 |
| unplaned interventions n(%) | 10(5.8) | 17(6.9) | 5(4.0) | 8(8.6) | 3（3.5） | 11(7.2) | 9(21.4) | 8(3.5) | 0.002 |
| LVOTS n (%) | 7(4) | 6(2.4) | 2(1.6) | 3(3.1) | 9（10.5） | 4(2.6) | 0(0) | 3(1.3） | 0.857 |
| RVOTS n (%) | 0(0) | 29(11.6) | 0 | 12(12.7) | 0(0) | 10(6.5) | 1(2.3) | 6(2.6） | 0.031 |
| AI≧3 n (%) | 0(0) | 0(0) | 4(3.3) | 2( 2.2) | 0(0) | 1(0.6) | 0(0) | 0 | 0.885 |
| PI≧3 n (%) | 0(0) | 32(12.8) | 0(0) | 3(3.1) | 0(0) | 5(3.2%) | 0(0) | 2(0.8） |  |
| MI≧3 n (%) | 3(1.6) | 3(1.2) | 0(0) | 0(0) | 1（1.1） | 3(1.9) | 2(4.6) | 5(1.3） | 0.478 |
| TI≧3 n (%) | 2(1.0) | 1(0.4) | 2(1.6) | 0(0) | 3（3.4） | 2(1.3) | 0(0) | 6(1.7) | 0.383 |
| NYHA n (%) |  |  |  |  |  |  |  |  | 0.000 |
| I | 166 (95.4) | 224 (91.8) | 113 (92.6) | 52 (59.8) | 73 (85.8) | 124 (82.1) | 26 (61.9) | 146 (65.1) |  |
| II | 3 (1.7) | 13 (5.3) | 6 (5.0) | 28 (32.2) | 9 (10.5) | 24 (15.9) | 5 (11.9) | 70 (31.2) |  |
| III | 2 (1.2) | 7 (2.8) | 3 (2.5) | 6 (6.9) | 3 (3.7) | 3 (2.0) | 10 (23.8) | 7 (3.1) |  |
| IV | 1 (0.6) | 2(0.8) | 0 | 1 (1.1) | 0 | 0 | 1 (2.4) | 1 (0.5) |  |
| primary endpoints n (%) n(%) | 3(1.7) | 7(2.8) | 8(6.5) | 9(9.6) | 6(7.0) | 11(7.2) | 5(11.0) | 12(5.3) | 0.022 |
| secondary endpoints n (%) | 14(8.1) | 24(9.7) | 13(10.6) | 18(19.3) | 9(10.5) | 23（15.2） | 14(33.3) | 21(9.3) | 0.000 |
| 5-y survival free from primary endpoint | 99.4% (98.2%-100%) | 97.9% (96.1%-99.7%) | 96.4% (92.9%-99.9%) | 93.4% (88.3%-98.5%) | 96.0% (91.5%-100%) | 94.5% (90.8%-98.2%) | 92.7% (84.7%-100%) | 95.2% (92.3%-98.1%) | 0.000 |
| 10-y survival free from primary endpoint | 98.7% (96.9%-100%) | 96.9% (94.6%-99.2%) | 92.1% (86.2%-98.0%) | 89.8% (82.9%-96.7%) | 89.8% (81.6%-98.0%) | 90.5% (84.6%-96.4%) | 85.8% (73.8%-97.8%) | 93.1% (89.0%-97.2%) | 0.000 |
| 15-y survival free from primary endpoint | 96.3% (91.4%-100%) | 96.9% (94.6%-99.2%) | 90.0% (82.9%-97.1%) | 87.7% (79.9%-95.5%) | 89.8% (81.6%-98.0%) | 90.5% (84.6%-96.4%) | 85.8% (73.8%-97.8%) | 93.1% (89.0%-97.2%) | 0.000 |
| 5-y survival free from secondary endpoint | 98.1% (96.1%-100%) | 97.5% (95.7%-99.3%) | 95.3% (91.4%-99.2%) | 88.7% (81.7%-95.7%) | 94.4% (88.3%-100%) | 91.8% (86.1%-97.5%) | 87.3% (76.1%-98.5%) | 96.3% (94.3%-98.3%) | 0.000 |
| 10-y survival free from secondary endpoint | 92.2% (86.0%-98.4%) | 92.1% (87.9%-96.3%) | 88.0% (80.8%-95.2%) | 78.1% (68.3%-87.9%) | 85.2% (73.8%-96.6%) | 82.7% (73.3%-92.1%) | 49.8% (24.7%-74.9%) | 89.3% (84.6%-94.0%) | 0.000 |
| 15-y survival free from secondary endpoint | 51.2% (8.9%-93.5%) | 27.5% (0.0%-68.8%) | 41.0% (0.0%-90.9%) | 66.5% (49.3%-83.7%) | 77.5% (60.7%-94.3%) | 78.6% (68.2%-89.0%) | 41.5% (12.8%-70.2%) | 83.7% (76.9%-90.5%) | 0.000 |

AV, LVOTS, left ventrcle outlet obstruction; RVOTS, right ventricle outlet obstruction; AI, aortic valve insufficiency; PI, pulmonary valve insufficiency; MI, mitral valve insufficiency; TI, tricuspid valve insufficiency; Other abbreviations as in Table 2.

**Supplemental Table 3 Survival Rates by Anatomical type According to the STS/EACTS Classification**

| Anatomical type (STS and EACTS) | 5y- survival (95%CI) | 10y- Survival (95%CI) | 15y- Survival (95%CI) |
| --- | --- | --- | --- |
| VSD type (n=180) | 99.4% (98.2%, 100%) | 96.3% (93.8%, 98.8%) | 95.0% (92.5%, 97.5%) |
| TOF type (n=256) | 97.9% (96.1%, 99.7%) | 96.9% (95.7%, 98.1%) | 94.5% (93.3%, 95.7%) |
| TGA type (n=241) | 95.1% (92.1%, 98.1%) | 89.0% (86.3%, 91.7%) | 87.0% (80.1%, 93.9%) |
| remote type (n=543) | 95.6% (94.4%, 96.8%) | 91.6% (89.5%, 93.7%) | 85.0% (81.0%, 89.0%) |

The 85 patients lost to follow-up were also included in the analysis, with their outcomes treated as censored. STS: The Society of Thoracic Surgeons; EACTS, The European Association for Cardio-Thoracic Surgery; VSD, ventricular septal defect; TOF, Tetralogy of Fallot; TGA, transposition of the great arteries.

**Supplemental Table 4 Survivals rates by each operational group**

|  | 5y-primary endpoint | 10y-primary endpoints | 15y-primary endpoints |
| --- | --- | --- | --- |
| BiV repair group | 97.5% [95.1%- 97.8%] | 94.2% [91.4%-97.0%] | 93.1% [89.7-，96.5] |
| SVP group | 93.4% [89.4%- 97.4%] | 91.6% [86.3%-96.9%] | 89.8% [83.2%- 96.4%] |
| palliation group | 88.9% [78.4%- 99.4%] | 80.9% [66.2%- 95.6%] | 72.9% [54.0- 91.8% |

Abbreviations as in Table 2. All the results was illustrated as Survival [95% CI].

**Supplemental Table 5 Cox proportional hazards regression model of the entire cohort**

|  | HR | lower .95 | | upper .95 | | *P* | |  |  |  |
| --- | --- | --- | --- | --- | --- | --- | --- | --- | --- | --- |
| age | 1.0 | | 1.0 | | 1.0 | | 0.812 |  |  |  |
| AV discordance | 0.5 | | 0.1 | | 1.5 | | 0.204 |  |  |  |
| CAVC | 2.0 | | 0.9 | | 4.2 | | 0.074 |  |  |  |
| Isomerism | 1.0 | | 0.3 | | 3.1 | | 0.989 |  |  |  |
| Heterotaxy | 1.3 | | 0.6 | | 2.6 | | 0.454 |  |  |  |
| VSD type |  | |  | |  | | 0.159 |  |  |  |
| TOF subtype | 1.6 | | 0.4 | | 6.3 | | 0.482 |  |  |  |
| TGA subtype I | 4.1 | | 1.1 | | 15.4 | | 0.039 |  |  |  |
| TGA subtype II | 5.0 | | 1.3 | | 19.7 | | 0.022 |  |  |  |
| remoted subtype I | 3.8 | | 0.9 | | 15.3 | | 0.064 |  |  |  |
| remoted subtype II | 3.5 | | 0.9 | | 13.5 | | 0.067 |  |  |  |
| remoted subtype III | 4.5 | | 1.0 | | 20.4 | | 0.052 |  |  |  |
| remoted subtype IV | 2.5 | | 0.6 | | 9.9 | | 0.201 |  |  |  |
| SVP group | 1.3 | | 0.6 | | 2.9 | | 0.514 |  |  |  |
| Palliation group | 3.1 | | 1.3 | | 7.3 | | 0.008 |  |  |  |

HR, Hazard Ratio; Other abbreviations as in Table 2.

**Supplemental Table 6 Fine–Gray Competing Risk Regression for Reoperation Risk**

| Variable | subHR | 95% CI | P-value |
| --- | --- | --- | --- |
| Age (per year) | 1.0 | 1.0 – 1.0 | 0.004 |
| Isomerism | 0.9 | 0.3 – 3.0 | 0.840 |
| Heterotaxy | 1.8 | 1.0 – 3.1 | 0.036 |
| AV discordance | 1.8 | 1.0 – 3.2 | 0.053 |
| CAVC | 1.7 | 0.8 – 3.7 | 0.150 |
| VSD type |  |  |  |
| TOF-type | 1.1 | 0.5 – 2.3 | 0.890 |
| TGA-subtype I | 0.8 | 0.3 – 2.1 | 0.600 |
| TGA-subtype II | 1.4 | 0.6 – 3.2 | 0.420 |
| remoted-subtype I | 0.6 | 0.2 – 1.8 | 0.330 |
| remoted-subtype II | 2.1 | 0.9 – 5.0 | 0.094 |
| remoted-subtype III | 1.7 | 0.8 – 3.7 | 0.170 |
| remoted-subtype IV | 1.2 | 0.5 – 3.2 | 0.650 |
| SVP vs. BiV | 0.2 | 0.1 – 0.6 | 0.002 |
| Palliation vs. BiV | 6.7 | 4.1 – 10.9 | <0.001 |

**subHR**: Subdistribution hazard ratio from Fine–Gray regression. Others abbreviations as in Table 2.

**Supplemental Figure legends**

**
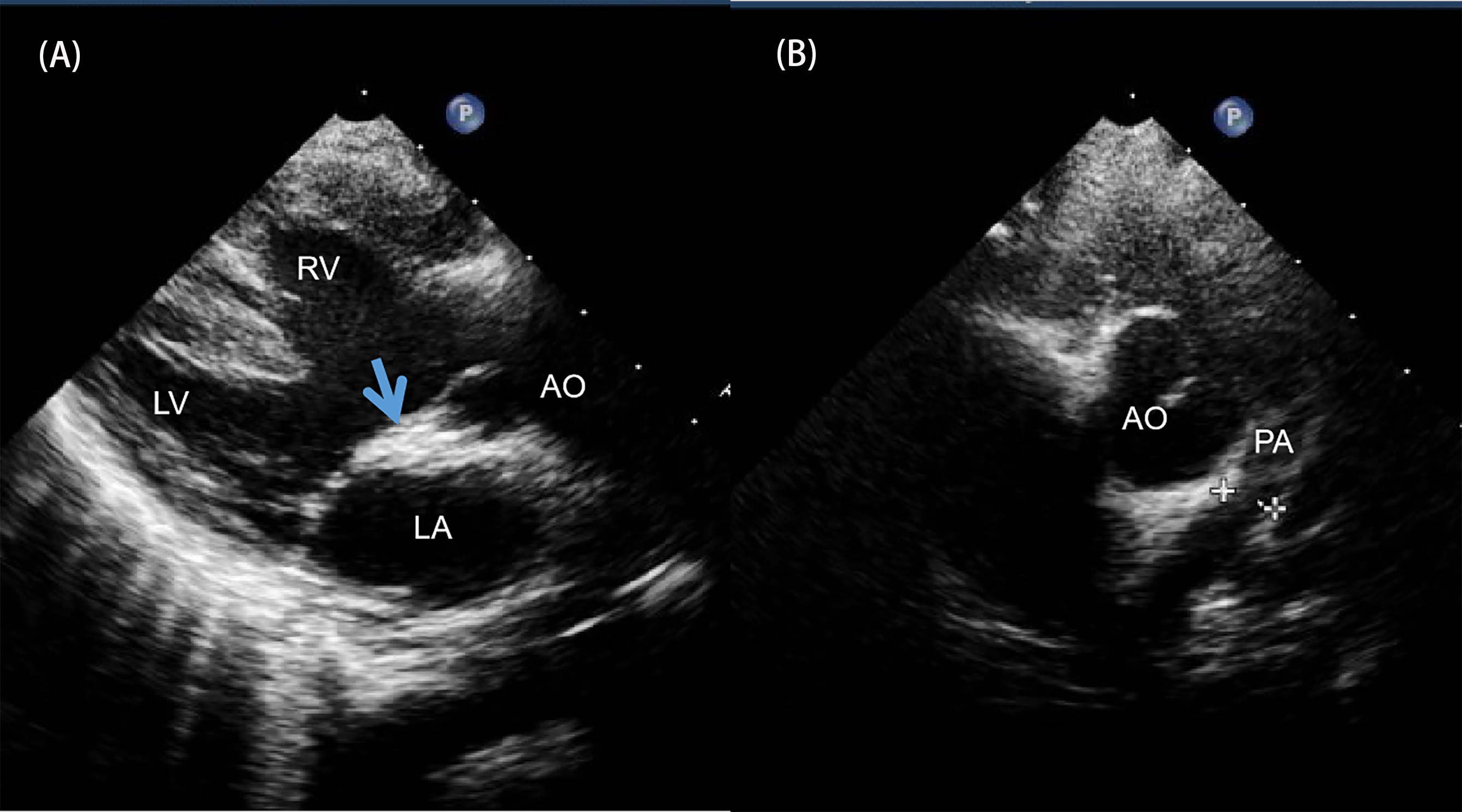
**

**Supplemental Figure 1** Echocardiographic findings in an anatomical subtype II patient. There is aortomitral discontinuity, and aortic override ≥50%, which is diagnostic for DORV.


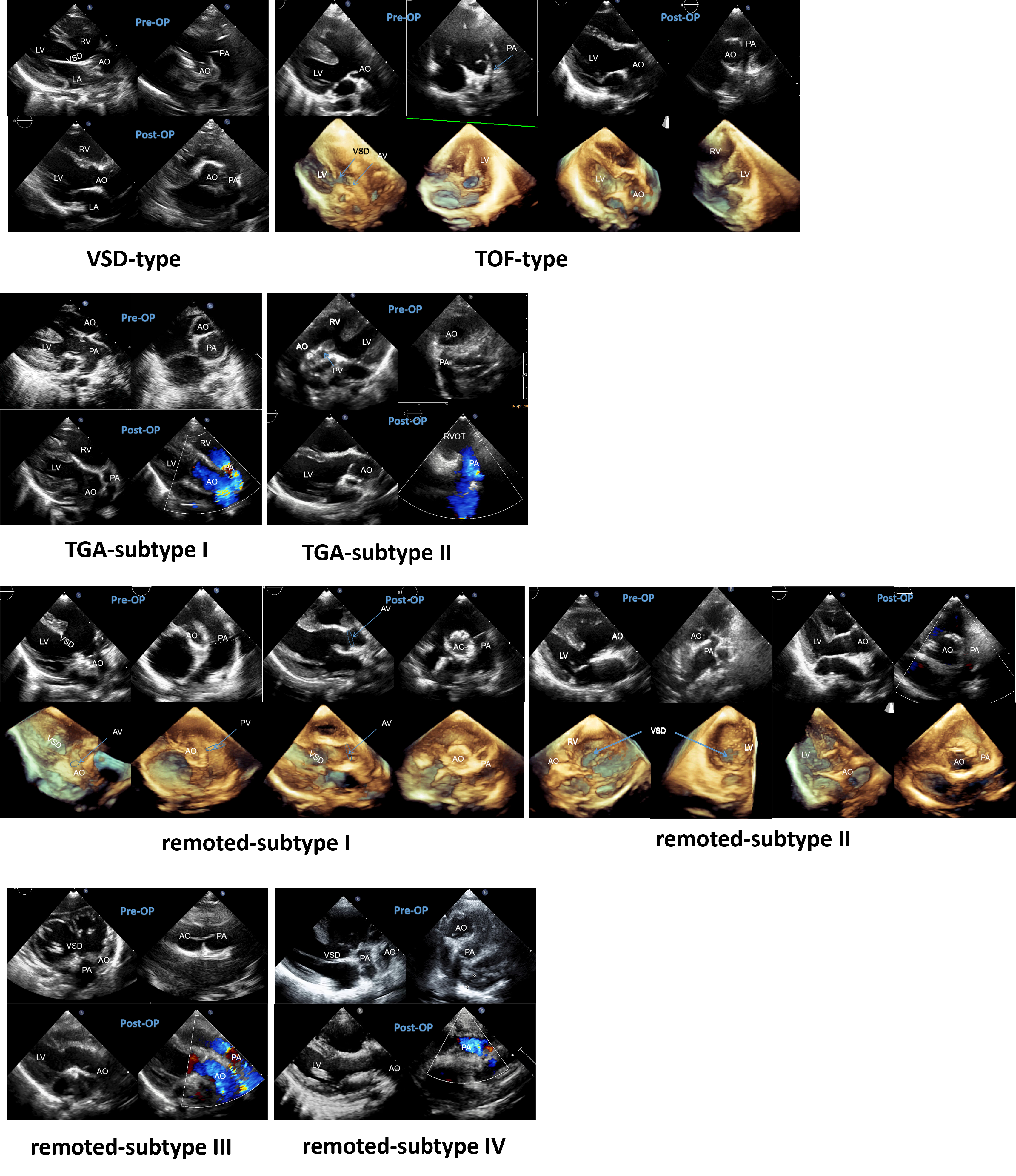


**Supplemental Figure 2** Pre- and postoperative echocardiographic images of all the anatomical subtype. OP, operation; LA, left atria; LV, left ventricle; RV, right ventricle; AO, aorta; PA, pulmonary artery; VSD, ventricular septal defect.


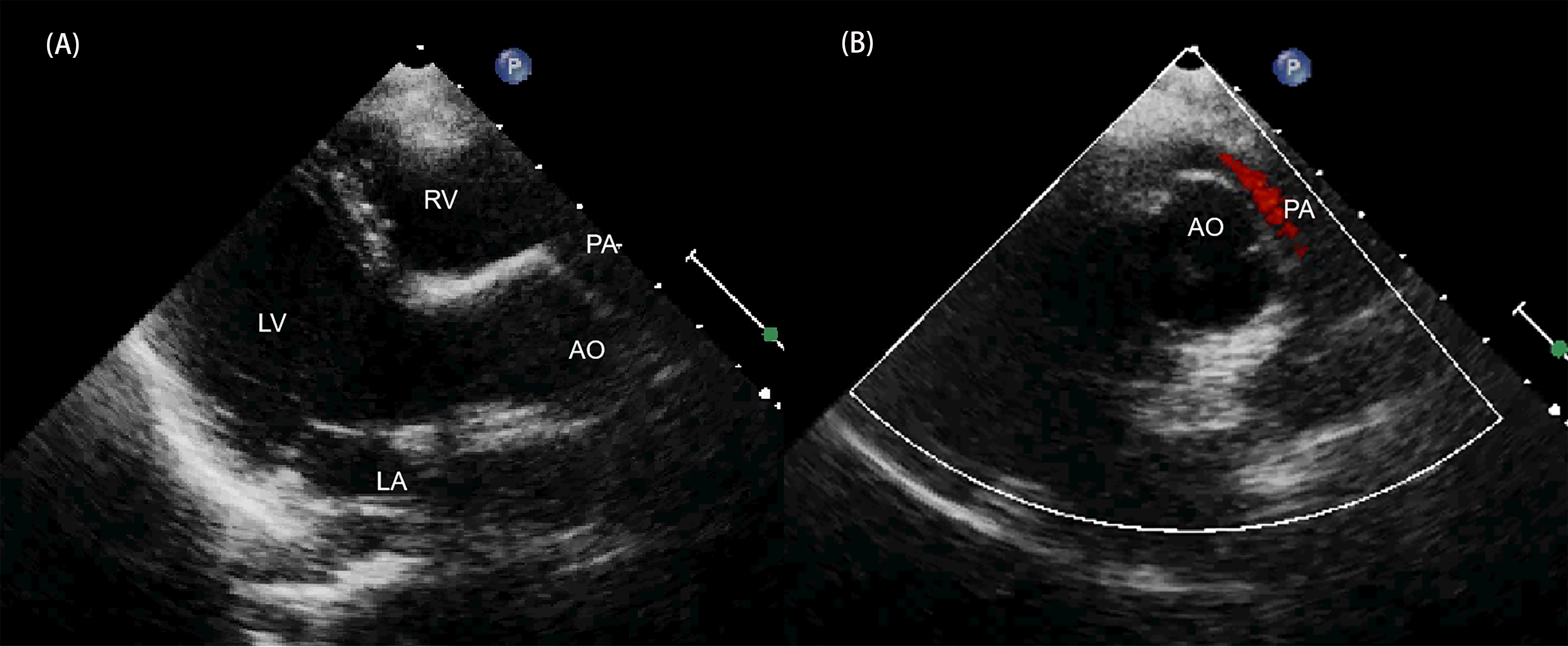


**Supplemental Figure 3** Postoperative echocardiographic images of a remote-subtype II patient who performed the Rev procedure.


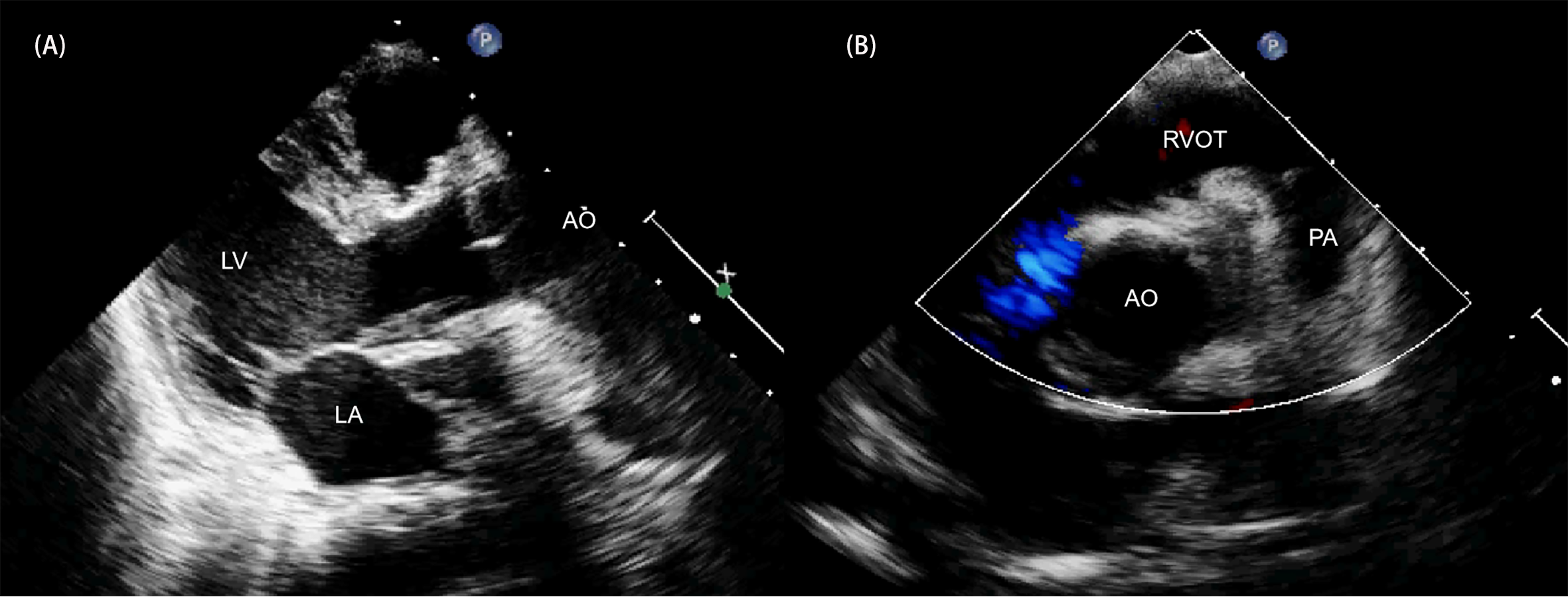


**Supplemental Figure 4** Postoperative echocardiographic images of a remote-subtype IV patient who performed the Ratelli with long intraventricular tunnel repair to the aorta procedure.


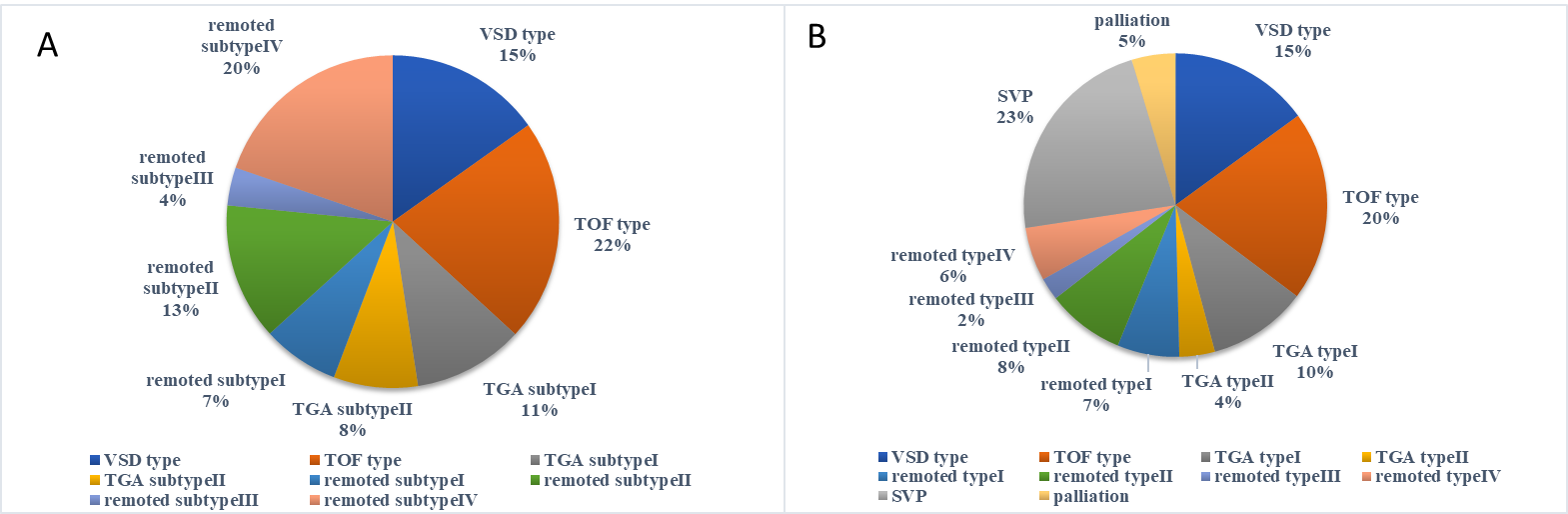


**Supplemental Figure 5** The distribution of anatomical subtypes (A) and operational types (B).


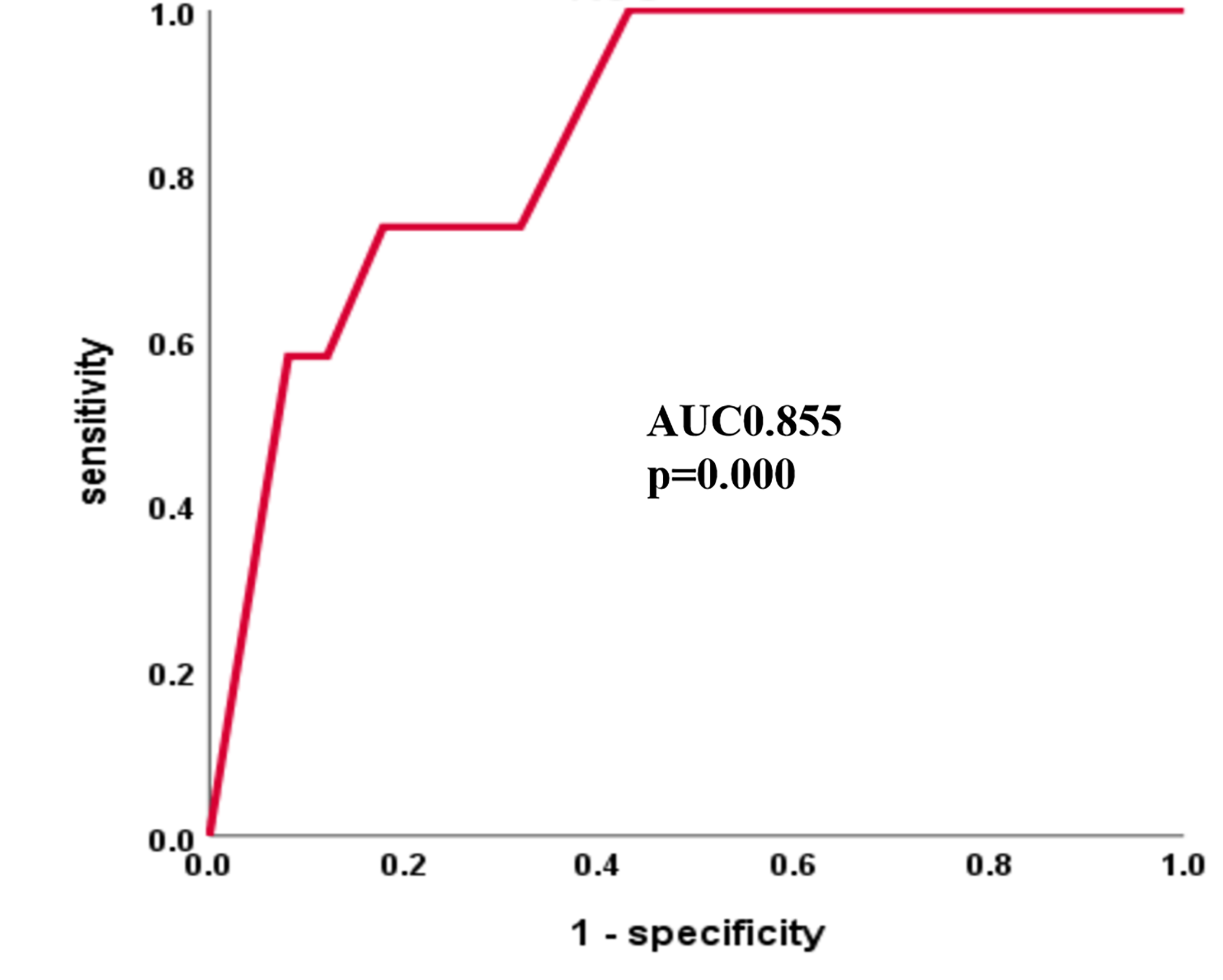


**Supplemental Figure 6** The ROC curve for predicting SVP procedure based on anatomical subtype.


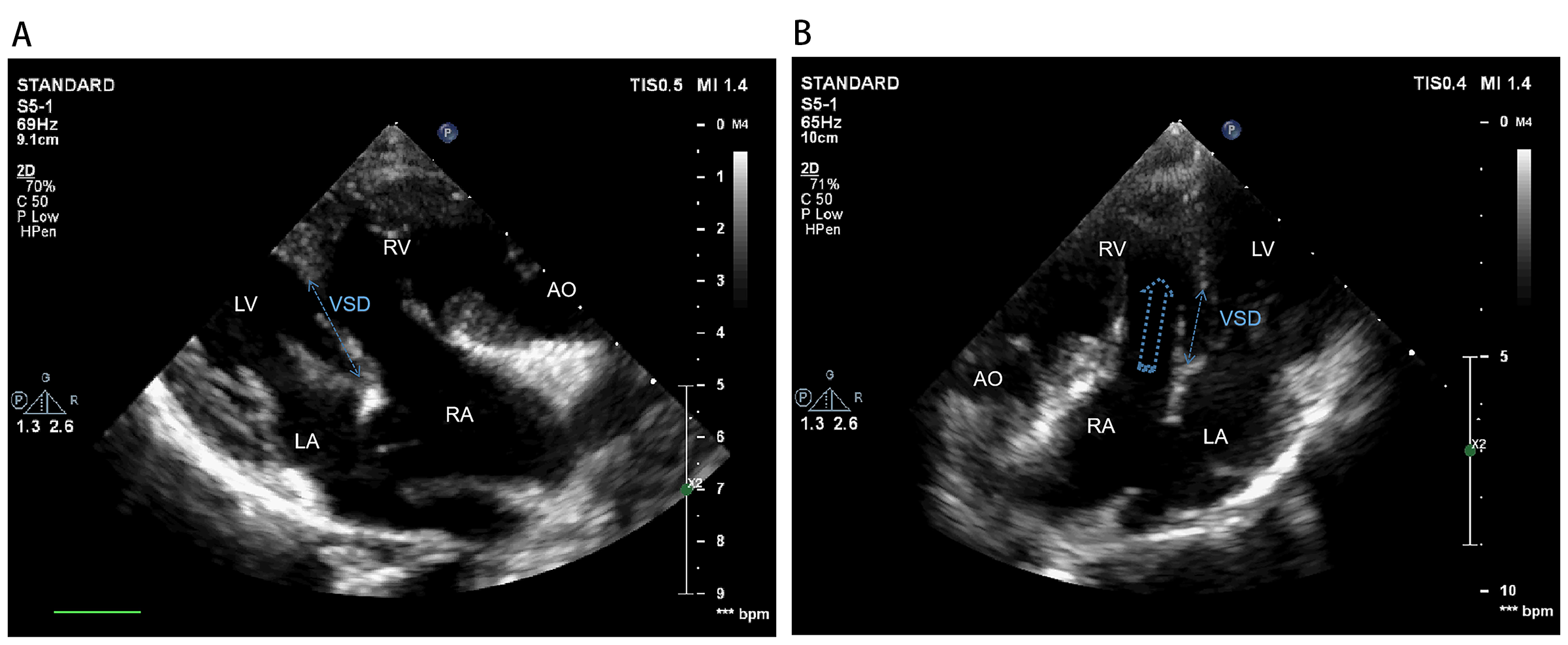


**Supplemental Figure 7** Echocardiagraphic image shows the location of VSD-NC make the planned long tunnel to arteries route through the tricuspid valve inflow tract, which prohibits a BiV repair. Parasternal Long-Axis Plane (A); Apical four chamber view (B). tricuspid valve inflow tract.

**Supplemental Video legends**

**Supplemental Video 1-I (1)**: Preoperative parasternal long-axis echocardiogram demonstrating VSD-type (corresponding to Supplemental Fig. 2-VSD-type (1)).

**Supplemental Video 1-I (2)**: Preoperative great artery short-axis echocardiogram demonstrating VSD-type (corresponding to Supplemental Fig. 2-VSD-type (2)).

**Supplemental Video 1-I(3)**: Postoperative parasternal long-axis echocardiogram demonstrating VSD-type (corresponding to Supplemental Fig. 2-VSD-type(3)).

**Supplemental Video 1-I (4)**: Postoperative great artery short-axis echocardiogram demonstrating VSD-type (corresponding to Supplemental Fig. 2-VSD-type

(4)).

**Supplemental Video 1-II (1)**: Preoperative parasternal long-axis echocardiogram demonstrating VSD-type (corresponding to Supplemental Fig. 2-TOF-type

(1)).

**Supplemental Video 1-II (2)**: Preoperative great artery short-axis echocardiogram demonstrating TOF-type (corresponding to Supplemental Fig. 2-TOF-type (2)).

**Supplemental Video 1-II (3)**: Preoperative 3D echocardiogram demonstrating TOF-type (corresponding to Supplemental Fig. 2-TOF-type (3)).

**Supplemental Video 1-II (4)**: Preoperative 3D echocardiogram demonstrating TOF-type (corresponding to Supplemental Fig. 2-TOF-type (4)).

**Supplemental Video 1-II (5)**: Postoperative parasternal long-axis echocardiogram demonstrating TOF-type (corresponding to Supplemental Fig. 2-TOF-type (5)).

**Supplemental Video 1-II (6)**: Postoperative great artery short-axis echocardiogram demonstrating TOF-type (corresponding to Supplemental Fig. 2-TOF-type (6)).

**Supplemental Video 1-II (7)**: Postoperative 3D echocardiogram demonstrating TOF-type (corresponding to Supplemental Fig. 2-TOF-type(7)).

**Supplemental Video 1-II (8)**: Postoperative 3D echocardiogram demonstrating TOF-type (corresponding to Supplemental Fig. 2-TOF-type (8)).

**Supplemental Video 1-III (1)**: Preoperative parasternal long-axis echocardiogram demonstrating TOF-type (corresponding to Supplemental Fig. 2-TGA-subtype I (1)).

**Supplemental Video 1-III (3)**: Postoperative parasternal long-axis echocardiogram demonstrating TOF-type (corresponding to Supplemental Fig. 2- TGA-subtype I (3)).

**Supplemental Video 1-III (4)**: Postoperative great artery short-axis echocardiogram demonstrating TOF-type (corresponding to Supplemental Fig. 2- TGA-subtype I (4)).

**Supplemental Video 1-IV (1)**: Preoperative parasternal long-axis echocardiogram demonstrating TGA-subtype II (corresponding to Supplemental Fig. 2- TGA-subtype demonstrating TGA-subtype II (1)).

**Supplemental Video 1-IV (2)**:pretoperative great artery short-axis echocardiogram demonstrating TGA-subtype II (corresponding to Supplemental Fig. 2- TGA-subtype demonstrating TGA-subtype II (2)).

**Supplemental Video 1-IV (3)**: preoperative 3D echocardiogram demonstrating TGA-subtype II (corresponding to Supplemental Fig. 2- TGA-subtype II (3)).

**Supplemental Video 1-IV (4)**: preoperative 3D echocardiogram demonstrating TGA-subtype II (corresponding to Supplemental Fig. 2- TGA-subtype II (4)).

**Supplemental Video 1-IV (5)**: Postoperative parasternal long-axis echocardiogram demonstrating TGA-subtype II (corresponding to Supplemental Fig. 2- TGA-subtype demonstrating TGA-subtype II (1)).

**Supplemental Video 1-IV (6)**: Post operative great artery short-axis echocardiogram demonstrating TGA-subtype II (corresponding to Supplemental Fig. 2- TGA-subtype demonstrating TGA-subtype II (2)).

**Supplemental Video 1-IV (7)**: Post operative 3D echocardiogram demonstrating TGA-subtype II (corresponding to Supplemental Fig. 2- TGA-subtype II (3)).

**Supplemental Video 1-IV (8)**: Post operative 3D echocardiogram demonstrating TGA-subtype II (corresponding to Supplemental Fig. 2- TGA-subtype II (4)).

**Supplemental Video 1-V (1)**: Preoperative parasternal long-axis echocardiogram demonstrating remote- subtype I (corresponding to Supplemental Fig. 2-remote- subtype I (1)).

**Supplemental Video 1-V (2)**: Preoperative great artery short-axis echocardiogram demonstrating remote- subtype I (corresponding to Supplemental Fig. 2- remote- subtype I (2)).

**Supplemental Video 1-V (3)**: Preoperative 3D echocardiogram demonstrating remote- subtype I (corresponding to Supplemental Fig. 2- remote- subtype I (3)).

**Supplemental Video 1-V (4)**: Preoperative 3D echocardiogram demonstrating remote- subtype I (corresponding to Supplemental Fig. 2- remote- subtype I (4)).

**Supplemental Video 1-V (5)**: Postoperative parasternal long-axis echocardiogram demonstrating remote- subtype I (corresponding to Supplemental Fig. 2 remote- subtype I (5)).

**Supplemental Video 1-V (6)**: Postoperative great artery short-axis echocardiogram demonstrating remote- subtype I (corresponding to Supplemental Fig. 2- remote- subtype I (6)).

**Supplemental Video 1-V (7)**: Postoperative 3D echocardiogram demonstrating remote- subtype I (corresponding to Supplemental Fig. 2- remote- subtype I (7)).

**Supplemental Video 1-V (8)**: Postoperative 3D echocardiogram demonstrating remote- subtype I (corresponding to Supplemental Fig. 2- remote- subtype I (8)).

**Supplemental Video 1-VI (1)**: Preoperative parasternal long-axis echocardiogram demonstrating remote- subtype II (corresponding to Supplemental Fig. 2- remote- subtype II (1)).

**Supplemental Video 1-VI (2)**: Preoperative great artery short-axis echocardiogram demonstrating remote- subtype II (corresponding to Supplemental Fig. 2- remote- subtype II (3)).

**Supplemental Video 1-VI (3)**: Preoperative 3D echocardiogram demonstrating remote- subtype II (corresponding to Supplemental Fig. 2- remote- subtype II (4)).

**Supplemental Video 1-VII (1)**: Preoperative apical five-chamber echocardiogram demonstrating anatomical subtype VII (corresponding to Supplemental Fig. 2- remote- subtype III (1)).

**Supplemental Video 1-VII (2)**: Postoperative parasternal long-axis echocardiogram demonstrating anatomical subtype VII (corresponding to Supplemental Fig. 2- remote- subtype III (3)).

**Supplemental Video 1-VII (3)**: Postoperative parasternal long-axis echocardiogram demonstrating anatomical subtype VII (corresponding to Supplemental Fig. 2- remote- subtype III (4)).

**Supplemental Video 1-VIII (1)**: Preoperative parasternal long-axis echocardiogram demonstrating remote- subtype IV (corresponding to Supplemental Fig. 2- remote- subtype IV (1)).

**Supplemental Video 1-VIII (2)**: Postoperative parasternal long-axis echocardiogram demonstrating remote- subtype IV (corresponding to Supplemental Fig. 2- remote- subtype IV (3)).

**Supplemental Video 1-VIII (3)**: Postoperative great artery short-axis echocardiogram demonstrating remote- subtype IV (corresponding to Supplemental Fig. 2- remote- subtype IV (4)).

**Supplemental Video 2A**: Postoperative echocardiographic images of a anatomic subtype VI patient who performed the Rev procedure (parasternal long-axis view, corresponding to Supplemental Figure 3A).

**Supplemental Video 2B**: Postoperative echocardiographic images of a anatomic subtype VI patient who performed the Rev procedure (great artery short-axis view, corresponding to Supplemental Figure 3B).

**Supplemental Video 3A**: Postoperative echocardiographic images of a anatomic subtype VIII patient who performed the Ratelli with long intraventricular tunnel repair to the aorta procedure (parasternal long-axis view, corresponding to Supplemental Figure 4A).

**Supplemental Video 3B**: Postoperative echocardiographic images of a anatomic subtype VI patient who performed the Rev procedure (great artery short-axis view, corresponding to Supplemental Figure 4B).

**Supplemental Video 4A**: Echocardiographic image shows the location of VSD-NC make the planned long tunnel to arteries route through the tricuspid valve inflow tract, which prohibits a BiV repair (parasternal long-axis view, corresponding to Supplemental Figure 8A).

**Supplemental Video 4B**: Echocardiographic image shows the location of VSD-NC make the planned long tunnel to arteries route through the tricuspid valve inflow tract, which prohibits a BiV repair. (apical four chamber view, corresponding to Supplemental Figure 8B).
